# Advances and Applications of Hybrid Graphene-Based Materials as Sorbents for Solid Phase Microextraction Techniques

**DOI:** 10.3390/molecules29153661

**Published:** 2024-08-02

**Authors:** Alessandra Timóteo Cardoso, Rafael Oliveira Martins, Fernando Mauro Lanças

**Affiliations:** Laboratory of Chromatography, Institute of Chemistry at Sao Carlos, University of Sao Paulo, P.O. Box 780, São Carlos 13566590, Brazil

**Keywords:** graphene-based materials, miniaturized sample preparation, stir bar sorptive extraction, microextraction by packed sorbent, pipette-tip solid-phase extraction, disposable pipette extraction, magnetic solid-phase extraction, green analytical chemistry

## Abstract

The advancement of traditional sample preparation techniques has brought about miniaturization systems designed to scale down conventional methods and advocate for environmentally friendly analytical approaches. Although often referred to as green analytical strategies, the effectiveness of these methods is intricately linked to the properties of the sorbent utilized. Moreover, to fully embrace implementing these methods, it is crucial to innovate and develop new sorbent or solid phases that enhance the adaptability of miniaturized techniques across various matrices and analytes. Graphene-based materials exhibit remarkable versatility and modification potential, making them ideal sorbents for miniaturized strategies due to their high surface area and functional groups. Their notable adsorption capability and alignment with green synthesis approaches, such as bio-based graphene materials, enable the use of less sorbent and the creation of biodegradable materials, enhancing their eco-friendly aspects towards green analytical practices. Therefore, this study provides an overview of different types of hybrid graphene-based materials as well as their applications in crucial miniaturized techniques, focusing on offline methodologies such as stir bar sorptive extraction (SBSE), microextraction by packed sorbent (MEPS), pipette-tip solid-phase extraction (PT-SPE), disposable pipette extraction (DPX), dispersive micro-solid-phase extraction (d-µ-SPE), and magnetic solid-phase extraction (MSPE).

## 1. Introduction

Since the confirmation of the existence of graphene (G), a single-layer carbon allotrope, in 2004 [1,2], this material has stood out in various areas, such as energy generation, electronics, sensors, and other material science fields, due to its unique properties, such as flexibility, lightness, thermal and electrical conductivity, and mechanical resistance to high pressures [3,4]. In addition to these, some peculiar characteristics of this nanomaterial, such as its extensive surface area, its binding sites arranged in a “honeycomb” pattern, the presence of delocalized π electrons, and its single-layer structure, among others, have prompted investigation of its use as a sorbent in sample preparation techniques, with the earliest records dating back to the early 2010s [5,6,7].

**Figure 1:**
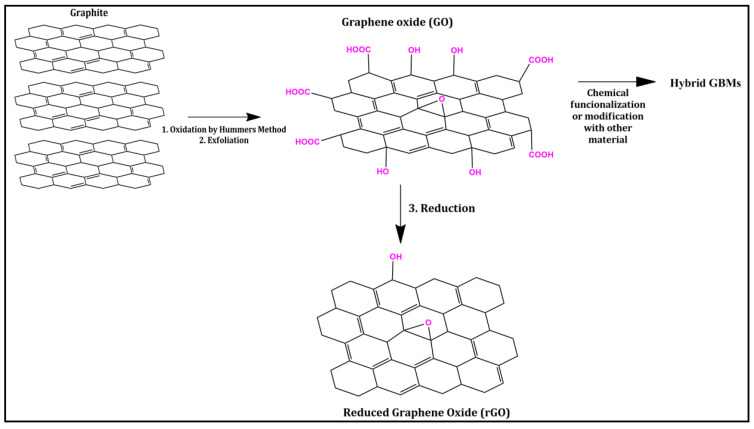
Scheme of obtaining GBMs from graphite oxidation.

Among the various methods for obtaining graphene, such as mechanical or chemical exfoliation from graphite, pyrolysis, and chemical vapor deposition (CVD), the most widely used method in the field of sorbent preparation is chemical synthesis or chemical reduction from graphene oxide (GO) [8]. GO is the precursor to graphene obtained from the oxidation of graphite, a step in which the Hummers methodology is often used and has undergone modifications over the years to improve the process. The single-layer structure of GO is very similar to that of graphene (Figure 1), except for the presence of oxygen-containing functional groups, such as epoxy, hydroxyl, and carboxyl, protruding from the nanosheet obtained through a change in the hybridization of carbon atoms from sp^2^ to sp^3^, imparting this derivative with a hydrophilic characteristic and enabling interaction with compounds containing more polar groups. Finally, after obtaining GO, a reducing agent is used to reduce the oxygenated groups of GO, resulting in reduced graphene oxide (rGO), which exhibits properties very similar to graphene, such as high hydrophobicity, albeit with different chemical structures [9].

In this context, especially in solid phase-based extraction techniques, there has been an increasing demand for sorbents with enhanced selectivity, i.e., those that promote efficient interactions with various analytes in the same sample, providing a less costly method aligned with the principles of green analytical chemistry (GAC) [10,11]. Thus, GO, in particular, allows for chemical functionalization with other components, such as ionic liquids (ILs) [12,13], silica derivatives [14,15], magnetic materials [16,17], molecularly imprinted polymers (MIPs) [18,19], resins [20], deep eutectic solvents (DESs) [21,22], and carbon-based biosorbents [23,24,25], among other materials. These materials are typically obtained through covalent bonding with oxygenated groups, resulting in hybrid materials promoting multiple interactions with the analytes and improving method performance, even in complex matrices [26].

Notably, modified graphene-based materials (GBMs) have been widely and successfully utilized in solid phase-based extraction techniques in conventional and miniaturized formats, with applications across various matrices, including biological, food, beverage, and environmental samples [27]. Their versatile applications underscore the importance of evaluating the methods’ efficiency, the phases’ selectivity, and the ecological effects they may cause. Several aspects, such as the toxicity of reagents and solvents used in synthesis and extraction methods, the quantity of sorbent and its potential for reuse, energy consumption, waste generation, sample consumption, and the volume of organic solvents used, need to be considered to minimize the environmental impact of each extraction process.

Following this brief introduction, this study aims to provide an overview of the literature on using modified GBMs in miniaturized solid-phase sample preparation techniques over the past seven years (from 2018 to May 2024). The first part of the article focuses on presenting the advances, types, and properties of hybrid GBMs, accompanied by a discussion of the environmental aspects of each material covered and their advantages and disadvantages. Some recent reviews have addressed the use of GBMs in extraction techniques, such as Jiang, Zhang, and Sun [27], who provided a comprehensive review of the use of graphene and graphene oxide in solid-phase extraction (SPE) and solid-phase microextraction (SPME) techniques, and Ibukun et al. [21], who compiled studies on the synthesis and characterization of graphene oxide modified with DESs for dispersive and magnetic solid-phase extractions. However, the existence of limited or outdated reviews on using hybrid GBMs in miniaturized extraction techniques suggests the need for additional studies to explore their potential in these approaches. Therefore, the second part of this review will focus on delineating recent applications specifically in miniaturized solid-phase sample preparation techniques, such as microextraction by packed sorbent (MEPS), pipette-tip solid-phase extraction (PT-SPE), disposable pipette extraction (DPX), dispersive micro-solid-phase extraction (d-μ-SPE), and stir bar sorptive extraction (SBSE), for the evaluation of different analyte(s) across a wide range of matrices. Finally, we present some of the current challenges and prospects related to the development and application of hybrid GBMs, providing a critique from the authors’ perspective.

## 2. Hybrid Graphene-Based Materials (Hybrid GBMs)

As discussed in the introduction, the presence of oxygenated groups in graphene oxide makes this material highly modifiable, driving the development of novel sorbent phases for various miniaturized sample preparation applications. Moreover, these materials have been proposed as low-cost and environmentally friendly alternatives to traditional sorbent phases for different sample preparation strategies [8]. This attribute stems from their biomass waste production, such as camphor leaves, wheat straw, and rice husks. These and other agricultural or forestry residues are rich in organic compounds like cellulose, hemicellulose, and lignin, which possess a high carbon content, thus making them suitable as precursors for the synthesis of GBMs via processes such as pyrolysis, CVD, and high-temperature carbonization (HTC), among others. Furthermore, due to their abundance and renewability, these materials typically incur lower costs than graphene synthesis from graphite and, depending on the production process, are also less detrimental to the environment [3,4]. Consequently, the following sections will provide a comprehensive literature overview detailing the main trends of materials anchored onto graphene and their utilization in diverse sample preparation methodologies. Table 1 shows some applications of hybrid GBMs that will be discussed in the following topics.

### 2.1. Silica Derivatives

The use of graphene derivatives in microextraction techniques is mainly due to their advantages, such as their high surface area, which allow these materials to be successfully employed in dispersive techniques. However, the irregular morphology of GBM particles is a challenge in terms of their use in techniques involving the packing of sorbents, such as automated in-tube solid-phase microextraction (IT-SPME) or online SPE, as well as offline techniques such as MEPS and PT-SPE, due to issues with high system pressure, device clogging, or particle aggregation. Functionalizing the oxygenated groups of GO or rGO with ionic liquids (ILs), deep eutectic solvents (DESs), carbon-based biosorbents, or magnetic particles can help resolve particle aggregation challenges, especially in dispersive techniques. However, in packed techniques, grafting GBMs onto the surface of the support material with precise and well-defined particles ensures better method performance, avoiding backpressure problems, and even improving the sorbent’s extraction capacity [46,47].

As a result, silica-derived materials, such as aminopropyl silica particles, are widely used to modify GBMs [8]. Maciel et al. [28] synthesized a sorbent based on graphene oxide supported on aminopropyl silica (GO@SiO_2_). They used it in a miniaturized extraction column within a column-switching method coupled with LC-MS/MS to extract and determine some β-lactam antibiotics from wastewater samples. The coupling between the aminopropyl silica and the graphene oxide occurred through covalent bonds between the carboxyl groups of the GO and the amino groups of the silica, using a water-based synthetic route, which is consequently more sustainable. The results showed that the method achieved recoveries above 60% for the analytes, demonstrating high performance due to using the prepared sorbent, which showed good stability and reusability for up to 100 cycles without significant losses in the extraction process. Previously, the same research group used GO@SiO_2_ to extract antidepressants and antiepileptics from urine samples in a capillary column in the first dimension of a multidimensional liquid chromatography system. In this study, low detection limits (LOD) for the analytes (0.5–20 µg L^−1^) were obtained, and the samples were analyzed without any prior treatment, such as precipitation and/or dilution. Additionally, the extraction column containing the modified graphene phase could be used more than 250 times, attesting to the robustness and stability of the sorbent [29]. 

**Figure 2:**
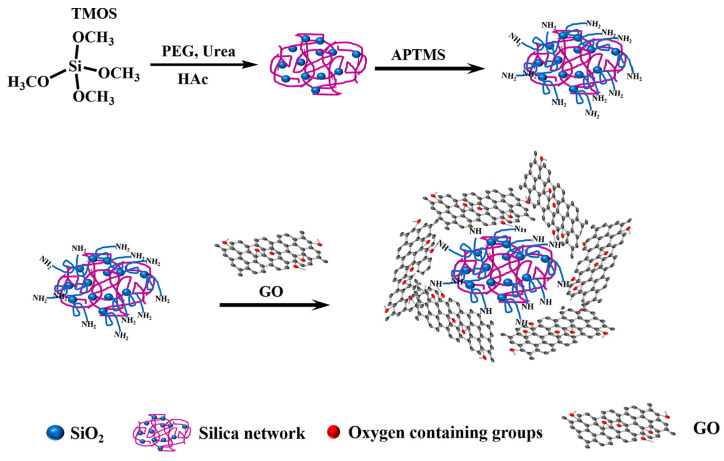
Schematic of preparation procedure of GO-anchored silica composite. Reproduced with permission [31] Copyright © 2024 Elsevier.

To enhance the selectivity of graphene particles supported on silica for a variety of compounds, in addition to the interactions promoted by the structure of GO and rGO (such as π-π and n-π interactions), hydrophobic interactions are further encouraged by functionalizing the graphene supported on aminopropyl silica with octadecylsilane (C18), thereby forming hybrid particles known as SiGO-C18 [47,48]. In this context, dos Santos et al. [30] employed this sorbent in an automated in-tube SPME method coupled with LC-MS/MS to quantify ten multi-class pesticides in Brazilian sugarcane spirits called “cachaças”. As a result, the authors demonstrated excellent analytical performance of the method, with accuracy percentages exceeding 80%, attributed to the interactions of the sorbent even with analytes of different physicochemical characteristics. More recently, Peng et al. [31] reported a sorbent with similar properties, consisting of GO anchored to silica through amino-terminated groups and doped with C18 applied in PT-SPE for the pre-concentration of aflatoxins in food, and analysis by liquid chromatography with fluorescence detector (LC-FLD) analysis. As the main advantage, the authors noted the appropriate polarity of the sorbent for the efficient enrichment of analytes compared to matrix compounds, achieving recovery rates greater than 70% for the four compounds studied and high precision between extractions. Figure 2 shows the process of obtaining GO particles anchored to silica.

Nonetheless, the support of graphene sheets on the surface of silica derivatives still allows for their functionalization with other compounds to further enhance these materials’ extraction capacity. The modification of Si@GO particles with β-cyclodextrin was addressed by da Silva et al. [23] to extract isoflavones from human urine. In their work, the authors reported using the sorbent as a coating in a “needle-sleeve” device in a fully automated solid-phase microextraction coupled with liquid chromatography (SPME-LC) system. The hybrid sorbent showed excellent performance for the intended purpose, attributed to the high stability and selectivity of GO and silica particles and both hydrophobic and hydrophilic characteristics of cyclodextrins. The authors highlighted the versatility of the sorbent by providing enhanced affinity with enrichment factors better than 3.8 times. The reported device could also be used more than 200 times while demonstrating adequate precision [23].

The studies mentioned in this topic demonstrate that incorporating graphene derivatives onto the surface of silica expands the application possibilities of these sorbents in various microextraction devices, especially in packing techniques (offline or online). Such a remarkable achievement is attributed to the improved mechanical resistance and morphology of the GBMs. Furthermore, the structural changes in these materials caused by these modifications result in the high stability and adequate reusability rates of these devices. Furthermore, the reusability of these materials significantly impacts green analytical practices since it reduces waste generation and contributes to the method’s sustainability, promoting a more environmentally friendly approach to sample preparation.

### 2.2. Ionic Liquids

ILs, composed of organic cations and inorganic anions, have been highlighted in sample preparation due to their unique properties, such as thermal and chemical stability, low volatility, and non-flammability [26,49,50]. Adjustable according to the choice of cations and anions, these liquids enhance the affinity and pre-concentration for various classes of analytes, improving sample cleanup through multiple interactions, including π-systems, hydrogen bonding, dispersive, electrostatic, and dipolar interactions [12,51]. 

In this regard, several studies have employed different classes of ILs in combination with other sorbent materials in microextraction techniques. They have been serving various roles, such as functional monomers, cross-linkers, porogenic solvents, coating materials, and, in some cases, even magnetic components, depending on their class [52]. Previously, ILs were used as alternatives to volatile organic solvents in liquid-liquid extractions (LLE) and elution solvents in SPE [49,50]. However, due to the toxicological potential of certain IL combinations and their persistence in aquatic and terrestrial environments, the use of small amounts of ILs as sorbent modifiers in solid-based sample preparation techniques appears to be a more environmentally viable option, as they are stable and highly reusable [50].

Nevertheless, functionalizing graphene derivatives with ILs makes the sorbent more stable. It helps to overcome problems such as the aggregation of its nanosheets, especially in dispersive extraction techniques in aqueous media. Furthermore, it increases the absorption capacity of the sorbent and reduces its loss during extractions [22,25,51]. Li et al. [13] developed an ionic liquid-thiol-graphene oxide composite (IL-TGO) that exhibited high extraction capacity for the pesticide fipronil, surpassing other commercial sorbents. The covalent functionalization of graphene oxide with 1-Allyl-3-pentafluorobenzyl imidazole bromide (PFBr) through intermediate thiol groups allowed hydrophobic and hydrogen interactions as well as the creation of new interaction sites through the IL. The stability of the sorbent enabled its reuse in over fifteen extraction cycles in chicken egg samples using a combined solid-phase microextraction technique. 

In addition to particulate sorbents, the use of ILs for monolith preparation has also been recently reported. Xiao et al. [25] prepared an aerogel using natural chitosan supported on GO layers through covalent bonds (IL–CS–GOA). Additionally, they added the ionic liquid—1-allyl-3-methylimidazolium tetrafluoroborate ([AMIm]BF_4_)—as a porogen and modifier, increasing the surface area of the sorbent, resulting in a porous three-dimensional aerogel for extracting polyhalogenated carbazoles from sediment samples. The authors reported that this combination of materials resulted in a high specific surface area of 173 m^2^ g^−1^, attributed to the addition of the IL. The sorbent was employed in a glass dropper extraction system, with just 8 mg showing a reusability rate of six cycles and recovery values above 80%, demonstrating its high adsorption capacity and efficient mass transfer for the analytes. In the field of magnetic ionic liquids (MILs), Oviedo and collaborators [32] described the synthesis of magnetized graphene oxide nanoparticles with trihexyl(tetradecyl)phosphonium hexachlorodysprosiate(III) ([P6,6,6,14]3DyCl6) (GO@MIL) sorbent, using the dispersive solid-phase microextraction technique (DμSPE) to extract inorganic antimony from water and food samples. The extraction efficiency for Sb(III) was over 99%, using only 3 mg of graphene oxide and 40 μL of MIL, resulting in a greenness metric for the method of 0.61, as confirmed by the Analytical GREEnness calculator (AGREE).

In this context, while the benefits of using ILs as modifiers and their associated adsorptive properties are increasingly recognized, evaluating sustainability metrics in both the preparation and use of these sorbents is essential. It is known that the toxic nature of an IL depends solely on its synthetic route and structure, steps such as single-stage synthesis reactions to avoid waste generation, careful selection of ions based on their toxicity to living organisms, and assessing sorbent reusability before method optimization can reduce environmental impact [26]. Additionally, new generations of ionic liquids, such as protic ionic liquids (PIL) formed by simple acid-base reactions and zwitterionic ionic liquids (ZILs), which feature functional ionic groups conferring a neutral overall charge, have been suggested as compounds with lower ecotoxicity compared to aprotic ionic liquids, thereby reinforcing their potential in more sustainable sample preparation practices [50,53,54].

### 2.3. Magnetic Materials 

The continual advancements in sample preparation within the scientific realm have paved the way for the introduction of magnetic nanoparticles (MNPs) or magnetic beads (MBs) as sorbent materials in countless miniaturized extraction strategies [55,56]. According to Vállez-Gomis [57], the magnetism inherent in these materials primarily stems from spinel ferrites (MFe_2_O_4_, where M represents Fe, Co, Mn, Ni, or Cu). Moreover, the authors assert that magnetic materials offer the advantage of being straightforward and cost-effectively synthesized, presenting remarkable application versatility. On the other hand, common drawbacks include their tendency to form agglomerates and undergo oxidation [56] quickly. Consequently, conventional strategies use inorganic protection or organic coatings to enhance their chemical stability [21,57]. Furthermore, given their active sites containing hydroxy, amino, or vinyl groups, the literature has increasingly demonstrated the potential functionalization of magnetic materials, particularly GBMs, for their application as sorbent materials in miniaturized extraction approaches [26,57]. 

The utilization of G in the synthesis of MNPs and MBs has showcased a remarkable synergy, leveraging the inherent properties of graphene. This synergy is particularly evident due to graphene’s robust π-π stacking interactions, mechanical durability, and thermal resilience [27]. Moreover, graphene-based magnetic materials are mainly fabricated by physical attachment or covalent binding [58]. The first approach is often regarded as the simplest, involving the straightforward mixing of graphene-based materials and MNPs either in solution or through the in-situ growth of MNPs. Conversely, covalent bonding entails an amidation reaction with crosslinking reagents, such as N-hydroxisuccinimide (NHS). Furthermore, the exploration of GO and rGO for their magnetization and application as sorbents in sample preparation techniques shows significant potential for their use as MNPs [57]. 

Most recently, Carvalho et al. [59] reported an ex-situ magnetization method for rGO material. The technique mainly involves the mixing and interaction of two previously prepared phases. The authors highlighted that, while the in-situ approach is commonly employed for producing graphene-based magnetic materials due to its one-step execution, it still suffers from limited control over reaction products and by-products. For the experimental part, the start rGO nanoparticles were immersed in a suspension of 1 mg mL^−1^ of Fe_2_O_4_-NPs for four days with later filtration and drying (50 °C). While the study highlights the significant potential for scalability, its drawbacks primarily stem from challenges related to the dispersion of nanostructures within the matrix. Therefore, future studies are needed to address this challenge effectively. Apart from this, an intriguing comparative study was proposed by Thy et al. [60], in which the authors conducted a comparison between iron magnetic nanoparticles-doped graphene oxide (Fe_3_O_4_-GO) synthesized via both in-situ and ex-situ approaches. The results primarily highlighted the larger specific area of Fe_3_O_4_-GO obtained by the in-situ approach compared to those from the ex-situ method. However, each approach’s advantages and drawbacks must be thoroughly evaluated before determining the optimal synthesis method for obtaining Fe_3_O_4_-GO sorbent materials.

Although in-situ approaches offer simplicity, the physical adsorption between magnetic materials and graphene-based nanoparticles may not ensure sufficient stability for repetitive use. In contrast, covalent bonding offers intriguing versatility in fabricating carbon-based MNPs. Li et al. [33] reported the development of graphene oxide-based magnetic covalent organic framework composites (GO@MCOFs). In their study, the MNPs were synthesized by grafting two monomers, tris(4-aminophenyl)amine, and 2,4,6-triformylphloroglucinol, onto Fe₃O₄ nanoparticles anchored on graphene oxide scaffolds. The reaction proceeded under continuous stirring for 2 h until the formation of the final composite material. Comprehensive characterization studies have revealed the resultant material exhibited significant paramagnetism and effective adsorption properties for the target analytes. While the covalent bonding approach may appear more laborious with more steps performed than the in-situ method, it holds immense potential for yielding more stable carbon-based MNPs. Hence, there is a crucial need for concerted efforts within the scientific community to explore and expand the applications of this synthesis approach.

The fabrication of graphene-based magnetic materials offers significant advantages over traditional materials, including high surface area, water solubility, and exceptional adsorption capabilities for target analytes. However, current challenges include the detachment of MNPs from the graphene surface and their tendency to agglomerate. Developing new synthesis strategies, such as functionalizing graphene magnetic nanoparticles and improving them with covalent bounding protocols, is crucial to addressing these issues. Moreover, from an environmental perspective, there is a pressing need to propose new green synthesis routes for producing these materials. Many existing synthesis protocols still rely heavily on large volumes of organic solvents. Achieving a balance between improving graphene-based magnetic materials and adopting eco-friendly synthesis protocols is essential to unlocking a new era of advanced materials.

### 2.4. Molecularly Imprinted Polymers

MIPs represent a distinct category of materials primarily utilized as sorbent phases in various conventional and miniaturized sample preparation methodologies. These materials are designed to facilitate selective interactions between the sorbent and the target analyte, thereby potentially enhancing the efficiency and specificity of the method. The synthesis of MIPs predominantly involves three common approaches: (I) non-covalent technique, (II) covalent pre-organized approach, and (III) semi-covalent strategy [61]. Across these synthesis methodologies, the fundamental principle remains consistent: employing a template-based approach wherein a template molecule, typically representing the target analyte, is utilized to construct a three-dimensional polymeric structure. The obtained structure contains specific cavities molded by the presence of the template molecule. Subsequent template removal through a washing procedure renders these cavities available for particular interactions with the target analyte, which shares analogous physicochemical properties with the template. This enables effective recognition and separation within complex matrices [52,61]. Given the inherent advantages of utilizing MIPs as sorbent phases, there has been a notable surge of interest in their combination with GBMs.

Yuan et al. [36] demonstrated the synthesis of a novel poly(deep eutectic solvents) surface-imprinted graphene oxide composite (PDESs-MIP/GO). The synthesis involved several distinct steps. Initially, sulfhydryl-functionalized GO (SH-GO) was prepared by adding (3-mercaptopropyl)trimethoxysilane to a GO dispersion. Subsequently, two DESs were synthesized. The first DES (DES 1), intended as the monomer for the final MIP, was created by mixing allyl trimethylammonium chloride and urea. The second DES (DES 2), used as the crosslinker, was synthesized by combining allyl trimethylammonium chloride and itaconic acid. The final PDESs-MIP/GO sorbent was formed using SH-GO as the carrier, DES 1 as the functional monomer, DES 2 as the crosslinker, and epinephrine as the template molecule. The resultant polymer exhibited a remarkable imprinting factor (~2.0), primarily attributed to the choice of starting materials. Moreover, this study highlights the potential of DESs in providing an eco-friendly alternative for synthesizing MIPs based on graphene oxide, effectively circumventing the use of traditional and harmful organic solvents typically employed in conventional MIP synthesis procedures.

Another modification of GO with MIP was proposed by Jian et al. [38]. In this study, the authors first modified GO using a polymerizable silane group to create reactive sites for polymer grafting. For the MIP synthesis, the molecularly imprinted polymer-graphene oxide (MIP-GO) was immersed in a solution containing triphenyl phosphate (TPhP) as the template molecule, acrylamide (AM), ethylene glycol dimethacrylate (EGDMA), and azobisisobutyronitrile (AIBN). Fibers were also prepared without the addition of GO. The main results demonstrated that the incorporation of GO significantly improved the physicochemical properties of the polymer, such as specific surface area and adsorption capability, highlighting the importance of including this graphene-based material to improve analytical aspects of MIP applications.

Chen et al. [39] proposed a similar MIP-GO using different organophosphate flame retardants (OPFRs) as the template molecules to evaluate their analytical performance. Among the synthesized polymers, the one with TPhP exhibited the highest imprinting factor value (10.3). Despite this, the authors introduced an innovative strategy by assembling the three synthesized fibers into a 250 μm o.d. array called the GO-based surface molecularly imprinted polymeric fiber array (GO/MIP-FA). This strategy demonstrated superior analytical performance compared to traditional fibers such as polydimethylsiloxane (PDMS) (100 μm o.d.) and polyacrylate (PA) (80 μm o.d.). The authors highlighted that traditional phases primarily cover the polarity scale but lack the specificity provided by the MIP-GO, underscoring the enhanced performance of their novel fiber array.

**Figure 3:**
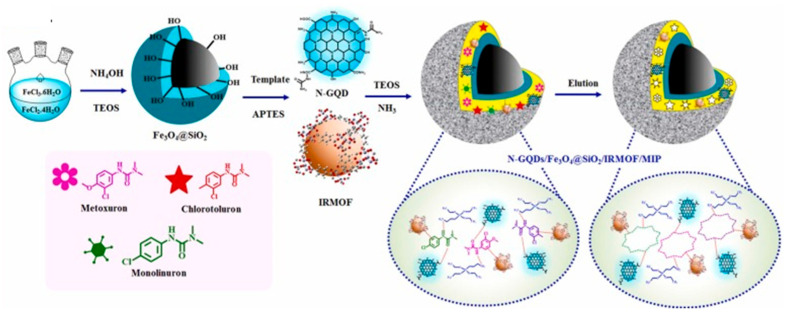
Schematic illustration of the N-GQDs/Fe_3_O_4_ synthesis approach @SiO_2_/IRMOF-1/MIP adsorbent. Reproduced with permission [37] Copyright © 2024 Elsevier.

A nitrogen-doped graphene quantum dots adsorbent was recently synthesized, integrating a zinc metal-organic framework and magnetic nanoparticles into a MIP framework (N-GQDs/Fe_3_O_4_ @SiO_2_/IRMOF-1/MIP) [37]. The hybrid material was crafted via the sol-gel method, employing motoneuron, chlorotoluron, and monolinuron as template molecules. Additionally, (3-aminopropyl)triethoxysilane (APTES) served as the functional monomer, while tetraethyl orthosilicate (TEOS) acted as the crosslinker for the MIP, N-GQDs, MNPs, and MOF components. The primary synthesis pathway of the hybrid material is depicted in Figure 3. Notably, the carbon-based sorbent exhibited a surface area featuring a mesoporous structure of 255.67 m^2^ g^−1^, surpassing the non-imprinted polymer (NIP) at 104.36 m^2^ g^−1^.

One of the notable advantages of MIPs, beyond their well-known selectivity, is their versatility in incorporating various sorbent materials for sample preparation methods. This section highlights that combining graphene-based materials with MIPs offers several analytical benefits, including significantly enhanced adsorption capabilities. Despite their unique features, a critical aspect of synthesizing graphene-based MIPs must be addressed. As for graphene-based magnetic materials, the synthesis of graphene-based MIPs often relies on traditional methods. This is particularly important because these protocols typically include a washing step to remove the template, which, depending on the affinity of the template molecule with the specific binding sites, can require a substantial amount of organic solvent. Various strategies have been explored in the literature to develop greener synthesis methods, such as using water and DESs [62]. These green strategies for graphene-based MIP synthesis can significantly reduce the environmental impact.

### 2.5. Graphene-Based Biosorbents

Aligned with green analytical chemistry (GAC) principles, utilizing biosorbents emphasizes employing renewable and biodegradable materials. This concept not only opens new avenues for advancing eco-analytical strategies but also underscores the significance of sustainability in analytical methodologies [63,64]. Furthermore, the integration of biomaterials, such as chitosan (CS) and cyclodextrins (CDs), offers additional benefits to GBMs, enhancing their versatility and efficacy across various complex matrices [65].

#### 2.5.1. Graphene-Based Materials Immobilized with Chitosan 

A significant challenge in utilizing GO, especially in water matrices, is the substantial aggregation stemming from π-π interactions among GO sheets and their inadequate dispersion in aqueous environments [66]. These drawbacks result in a considerable decline in adsorption efficiency by constricting the accessible surface area for molecular interaction. To address these challenges, using CS is a promising strategy for immobilizing graphene-derived materials [66,67]. CS, a biopolymer endowed with notable biodegradability, non-toxicity, and intriguing physicochemical attributes, presents a compelling solution for graphene-based materials. Its functional groups, including -OH and -NH_2_, facilitate the activation of adsorption sites while enhancing electrostatic interactions and hydrogen bonding with functional groups inherent in GO, for example [66,68]. Despite the CS’s intrinsic limitations in thermal stability and mechanical properties, these shortcomings are mitigated through synergistic interactions upon integration with graphene-based materials. Consequently, CS serves as a stabilizing agent for GO sheets, effectively mitigating aggregation issues [66].

Graphene oxide/chitosan (GO/CS) immobilized sorbents are synthesized through various methods, including sol-gel [69] processing and chemical crosslinking [70]. However, the conventional approach typically entails dissolving chitosan in an acetic acid aqueous solution (1–3% *v*/*v*). This process yields a yellowish-colored homogeneous solution obtained from the solubilization of chitosan. Moreover, the solution of graphene-based material is combined with the CS solution, followed by sonication until a homogeneous mixture is achieved. This amalgamation forms the basis of the GO/CS material, which can be utilized directly or subjected to subsequent processes, such as freeze drying [66]. Due to its inherent simplicity, this synthesis procedure has been widely used to produce various sorbent materials, primarily for application in microextraction protocols, as discussed in this section.

Peng et al. [40] reported the fabrication of a GO/CS aerogel for the extraction of hydrophobic pollutants. The authors employed a blend of ethanol and the cross-linking agent terephthalaldehyde in their synthesis approach, followed by vacuum freeze-drying for 24 h. Key findings underscored the superior analytical performance of the resultant GO/CS sorbent compared to conventional sorbent phases, such as polydimethylsiloxane/divinylbenzene (PDMS/DVB). This enhancement was primarily attributed to the robust hydrophobic, π-π, halogen bond, and hydrogen bond interactions between the coating and the analytes. While using cross-linkers in the synthesis of GO/CS has garnered considerable attention, conventional procedures outlined in the literature remain relevant. Ayazi, Saei, and Sarnaghi [71] demonstrated the synthesis of GO/CS material via the traditional method, as previously described, followed by filtration and drying steps.

In pollutant detection, incorporating cross-linking agents into GO/CS sorbent materials has been extensively documented in the literature for their significant impact on the abundance of functional groups available for binding with these compounds. As has been previously reported by Alves et al. [66], the authors stated that the hydrolysis reaction of borax leads to the formation of tetrahydroxyborate ions B(OH)^4−^ upon interaction with the -OH groups inherent in CS and GO. This reaction results in the formation of orthoborate chemical bonds, thereby exerting a notable influence on the ultimate adsorption characteristics of the GO/CS material. As previously mentioned, Peng et al. [40] the use of terephthalaldehyde as a cross-linking agent. According to the authors, this cross-linking agent was crucial for ensuring the stability of the GO/CS biosorbent in the SPME fibers. Cross-linking agents can be a valuable strategy to enhance the stability of sorbents in microextraction procedures, as these protocols often involve fragile systems. By incorporating cross-linkers, the durability and performance of the sorbents in microextraction applications can be significantly improved.

The cross-linking process has garnered significant attention in the literature, aiming to decipher its true implications for the ultimate efficacy of graphene-based chitosan biosorbents [72]. Given its pivotal role in determining the abundance of functional groups primed for binding with target analytes, the timing of this step holds considerable sway over the resultant polymer composition [66]. Despite this, different protocols that do not incorporate cross-linking in their synthesis have been reported. An interesting example is provided by Ghani et al. [41] who demonstrated the synthesis of a graphene oxide-coated agarose/chitosan (ACGO) biosorbent. The biosorbent was obtained through a simple freeze-drying procedure involving a mixture of chitosan and agarose with graphene oxide. Remarkably, the resulting polymer exhibited adequate mechanical structure and stability without cross-linking treatment.

#### 2.5.2. Graphene-Based Materials Immobilized with Cyclodextrin

CDs are oligopolysacchrides typically derived from starch through the enzymatic action of cyclodextrin glucanosyl transferase [24]. These molecules boast a distinctive structure characterized by a hydrophobic inner cavity and a hydrophilic surface, imparting remarkable physicochemical properties [73,74]. Considering their sample preparation application, one of the main advantages of CDs is their unique shape and functionalization potential. Werner et al. [65] CDs exhibit a cone-like structure reminiscent of hollow truncated cones, facilitating the encapsulation of guest molecules within their framework. Furthermore, the abundance of hydroxyl groups renders CDs ideal candidates for functionalization with various materials, enabling the creation of tailored sorbent phases [75]. 

When considering their role as sorbent phases, covalent bonding to solid supports bolsters the stability of cyclodextrins against water solubility challenges. Consequently, strategies for developing CD-based sorbents predominantly involve (I) immobilization onto inert supports, (II) integration with diverse nanomaterials, and (III) formation of nanosponges (NSs) through polymerization of cyclodextrins with an appropriate cross-linking agent [75,76]. Regarding item II, coupling cyclodextrins with graphene-based materials has showcased significant promise in forging novel biosorbent phases. This combination amplifies the utility of graphene-based phases and expands their applicability across a spectrum of analytical strategies and complex sample evaluations [8]. 

The literature reports many synthesis approaches for obtention cyclodextrins with carbon-based materials. Ning et al. [42] detailed the synthesis of a magnetic graphene oxide sorbent functionalized with β-cyclodextrin (NiFe_2_O_4_@GO@β-CD), employed for the magnetic solid-phase extraction of bisphenols. In their method, the previously fabricated NiFe_2_O_4_@GO phase was combined with β-CD to create the ultimate sorbent phase. This resultant product showcased a substantial increase in specific surface area, attributed to the incorporation of β-CD, which enhanced the adsorption of the target analyte by host-guest interactions, such as hydrogen bonding and π-π interactions. Hybrid materials based on β-CD and GO have also been introduced to enhance the applicability of these materials and propose different synthesis strategies for their obtention. For example, Silva and Lanças [24] reported the synthesis of β-cyclodextrin coupled to graphene oxide supported on aminopropyl silica (β-CD@GO@Sil). This synthesis involved using APTES combined with β-cyclodextrin for a subsequent reaction with graphene oxide and aminopropyl silica. The results showed that the final β-CD@GO@Sil material exhibited the adsorption characteristics typical of graphene-based materials. Furthermore, the selectivity of the material was attributed to the inclusion of β-cyclodextrin. The authors highlighted that supporting the material on aminopropyl silica opens new and unexplored horizons for applying graphene biosorbents in microextraction protocols.

Wang et al. [43] introduced an NS composed of polydimethylsiloxane/graphene oxide/β-cyclodextrin (PDMS/GO/β-CD) as a solid sorbent phase for detecting lavender essential oil. The fabrication of this NS involved three primary steps: preparing the PDMS sponge, modifying it with GO, and subsequently functionalizing it with β-CD. In the penultimate steps, the polydimethylsiloxane/graphene oxide (PDMS/GO) sponge was derived through the amidation process of graphene oxide with polydimethylsiloxane/polydopamine/3-aminopropyltriethoxysilane (PDMS/PDA/APT), followed by solvent washing. Subsequently, the PDMS/GO/β-CD composite was obtained by functionalizing the PDMS/GO sponge with β-cyclodextrin, after which it was allowed to dry at 60 °C to achieve the final NS sorbent. The results revealed a hierarchical porous interconnected morphology featuring continuous macro-sized cavities, enabling the material to be utilized in six sorption-desorption cycles while maintaining satisfactory analytical performance. 

While CS and CD are widely recognized as two of the most commonly utilized biopolymers for graphene derivative modification, the literature also highlights alternative methods for crafting graphene-based biosorbents, particularly for application in microextraction procedures. For instance, cellulose has proven to be an excellent material for modification with graphene-based sorbents. Recently, Wang et al. [77] described the synthesis of a reduced graphene oxide/cellulose nanocrystal (rGO/CNC) composite for microextraction applications. The biosorbent was produced by mixing GO and cellulose in a reaction medium, followed by a hydrothermal reaction and a freeze-drying process. Furthermore, agarose [41] and alginate [78] biopolymers have emerged as viable candidates for crafting graphene-based materials, showcasing the adaptability of graphene in manifesting diverse modification potentials with these natural materials.

CS and CD have found widespread application in conjunction with GBMs, leveraging their unique attributes such as remarkable supramolecular recognition and facile functionalization. Indeed, the exploration extends beyond CS and CD; various other natural materials have been investigated, broadening the spectrum of synthesis approaches and material choices for diverse applications. Furthermore, it is equally important to note that utilizing biosorbents based on GBMs represents a significant advancement towards environmentally conscious practices in analytical chemistry. Due to their inherent biodegradability, synthesis procedures employing GBMs with biomaterials are environmentally friendlier than traditional methods. As a result, this approach protects the environment from chemical waste production and ensures the well-being of researchers interested in applying such materials.

### 2.6. Deep Eutetic Solvents

Since the discovery of the adsorptive properties of GBMs and their utilization in sample preparation techniques, various materials with unique properties have been reported as functionalizers aimed at enhancing the extraction capacity of these sorbents, as discussed in the sections of this topic. However, some of these materials have a significant drawback: the use of toxic organic reagents or solvents in their preparation and an additional purification step. Therefore, the quest for more sustainable materials has brought DESs into the spotlight for modifying GBMs [21]. DESs, such as ILs, are designer solvents, meaning their physicochemical properties can be tailored according to their compounds, which are formed from eutectic mixtures of two or more components, one acting as a hydrogen bond donor (HBD) and the other as a hydrogen bond acceptor (HBA) through non-covalent interactions, resulting in compounds with melting points < 100 °C. Additionally, their preparation is carried out in just one step and does not require a purification step [79,80]. Their properties include thermal stability, low vapor pressure, low flammability, low volatility, adjustable composition, high solvation capacity, and higher biodegradability rates.

Additionally, they are easy to obtain and synthesize, low-cost, and highly reusable, and their constituents are derived from renewable sources such as alcohols, amines, carboxylic acids, sugars, glycols, phenols, quaternary ammonium salts, and phosphonium salts. These qualities make DESs a greener and safer alternative than ILs [79,81]. Various applications have been reported in modifying GBMs, with investigations in diverse areas such as food, beverages, environmental, and biological samples [22,44,45,82]. 

Miyardan et al. [22] developed a sorbent based on GO nanoparticles modified with a DES consisting of phosphocholine chloride/1-naphthol for pesticide residue extraction in zucchini using an approach combining d-μ-SPE combined with dispersive liquid-liquid microextraction (DLLME). The authors reported that GO was modified by simple ultrasonication, and its adsorption capacity was enhanced due to the presence of DES compared to unmodified GO, with recovery values above 70% for the eight pesticides investigated. In the field of hydrophobic DESs, Yuan et al. [44] modified GO with allyltriethylammonium bromide/ethylene glycol to extract toluene and xylene exposure biomarkers in urine samples by PT-SPE. The selectivity of the prepared sorbent, deep eutectic solvent functionalized graphene oxide (DFG), proved superior to some evaluated commercial phases and even pure GO for extracting the same analytes. This fact was attributed to functional groups in the DES, which promote multiple adsorption mechanisms with the biomarkers, including hydrophobic interactions. Additionally, 2 mg of the sorbent in the extraction device recovered percentages above 90% of the analytes. 

**Figure 4:**
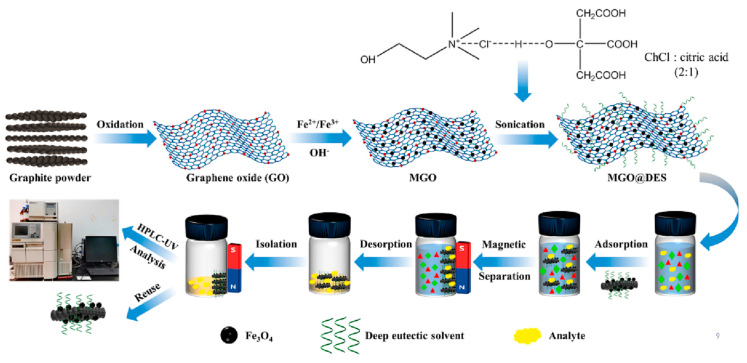
Schematic diagram of the MGO@DES and MSPE procedure. Reproduced with permission [45] Copyright © 2024 Elsevier.

Hao et al. [45] developed a magnetic graphene oxide based on Fe_3_O_4_ modified with choline chloride/citric acid, resulting in a hybrid material (MGO@DES) used in magnetic solid phase extraction (MSPE) for the extraction of three estrogens in milk samples (Figure 4). In addition to the π-π and hydrogen interactions already promoted by GO, the hydrophobicity of the sorbent was enhanced by the addition of DES, which showed excellent selectivity for more non-polar analytes through the increase in hydrophobic interactions, as well as electrostatic interactions promoted by the opposite charges of the analyte and the sorbent in the extraction medium. The method showed recovery rates above 90% for the investigated estrogens using only 3 mg of sorbent and the possibility of reuse for seven cycles without considerable loss in extraction efficiency.

Therefore, as observed in this topic, DESs can be tailored according to the desired interaction mechanism. When coupled with GBMs, they can extract various types of analytes, even in complex samples. Moreover, the modification of these sorbents also addresses the issue of aggregation, particularly in dispersive techniques, where the sorbent needs to be homogeneously dispersed to achieve extraction efficiency [44,82]. Consequently, their ecological advantages and ease of obtainment make methods more environmentally friendly, rendering them promising in sample preparation, specifically in functionalizing GBMs.

The synthesis of GBMs has demonstrated versatile approaches for obtaining enhanced sorbent materials. These materials possess remarkable physicochemical properties, showcasing the great potential of GBMs in creating hybrid and superior sorbents compared to traditional ones. However, significant effort is still needed to propose green synthesis approaches for these materials, as many current methods are not environmentally friendly. The use of biodegradable materials and reagents can overcome the main drawbacks of non-eco-friendly synthesis. This is particularly important given the literature highlighting the risks of toxicity to living organisms and the environment. These materials have been noted for their potential to penetrate cellular structures through various exposure routes [83]. According to Ghulam et al. [84], factors such as lateral size, surface structure, functional groups, purity, dosage, and exposure time influence the toxicity of these materials. Therefore, green analytical practices must include the conscious use and production of these materials, along with adequate synthesis conditions, to avoid human and environmental risks. The application of green alternatives for obtaining GBMs has the potential to expand their applications across different fields, enhancing their practical uses. 

## 3. Selected Applications of GBMs in Key Miniaturized Techniques

**Figure 5:**
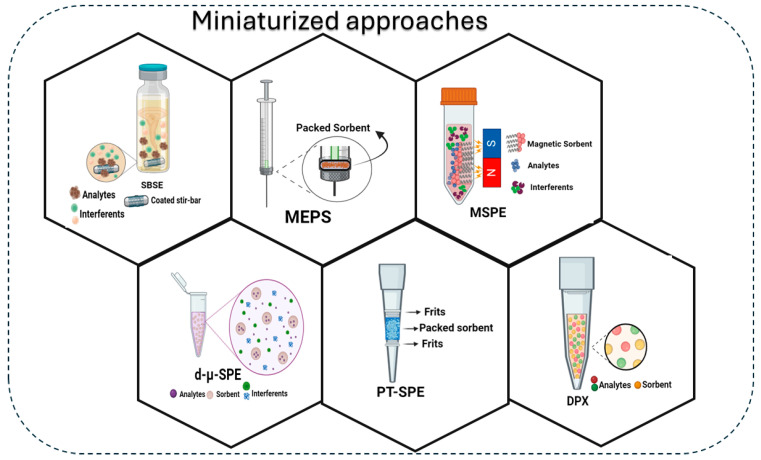
Miniaturized offline sample preparation techniques employing hybrid GBMs.

As demonstrated earlier, combining GBMs with various materials can produce a multitude of new sorbents with unique physicochemical properties and enhanced selectivity, thereby expanding their utility for diverse applications. Figure 5 illustrates the miniaturized sample preparation approaches in which graphene compounds have been a growing trend in hybrid GBMs. Therefore, this section will discuss some applications of graphene-based sorbents in different matrices for SBSE, MEPS, PT-SPE and DPX, d-µSPE, and MSPE, miniaturized methods.

### 3.1. Stir Bar Sorptive Extraction 

SBSE is a miniaturized technique that emerged in 1999. It is based on using a solid phase employed on a magnetic stirring bar as a device for compound extraction. This technique operates on the principle of partitioning, making it a non-exhaustive technique, in contrast to SPE. The extraction occurs with the exposition of the device containing the extracting phase to the sample medium for a certain period under constant agitation until the partition equilibrium of analytes between the solid and liquid phases is reached. Subsequently, this magnetic bar is exposed to an organic solvent that has an affinity for the compounds adsorbed on the bar, allowing the migration and subsequent injection of this extract into the analytical system [85]. Various coating materials for the bars have been investigated and reported in the literature since its inception, and in the field of graphene derivatives, their applications are diverse [86]. 

In the environmental field, Zhang et al. [87] used a porous nickel foam modified with reduced graphene oxide on its surface (rGO-NF) as a stirring bar for the extraction of six benzotriazole ultraviolet absorbents (BZTs) and their determination by liquid chromatography with a diode array detector (LC-DAD). One of this study’s advantages was the extraction device’s cost-effective preparation, as the authors reported acquiring the nickel foam commercially, and the hydrothermal reduction of GO occurred in situ on the substrate surface. Additionally, the method demonstrated enrichment factors exceeding 30% for the proposed method, with LODs in the range of 0.33–0.50 μg L^−1^ for the studied compounds. The device could be reused approximately 25 times without a loss in extraction efficiency, showcasing the significant stability of the rGO coating. Another study focused on developing a new coating for stirring bars based on a zirconium dioxide-reduced graphene oxide nanocomposite (ZrO_2_-rGO). The authors used the sol-gel methodology to coat the extraction bar made of a magnet encapsulated in glass and its application for the extraction of the organophosphate pesticide ethion in agricultural and river wastewater samples, followed by analysis using negative corona discharge ion mobility spectrometry (NCD-IMS). The method demonstrated a LOD of 1.5 μg L^−1^ and recoveries above 90% for the pesticide in actual samples. Furthermore, the ZrO2-rGO-coated bar showed higher sorption efficiency for ethion compared to a bar coated with polydimethylsiloxane (PDMS), attributed to the greater polarity of ZrO_2_-rGO, which enhances affinity interactions with the pesticide [88].

Another approach to this method is stir bar sorptive-dispersive microextraction (SBSDME), which combines the advantages of SBSE and d-µSPE. This method utilizes sorbents based on magnetic nanoparticles that dynamically coat an apparatus containing a bar-shaped magnet. When the device is introduced into the sample at a low stirring speed, the particles remain attached to the magnet, operating like SBSE. As the speed increases, the coating material disperses in the sample due to rotational force. At the end of the procedure, when the rotation stops, the magnetic particles reassemble on the magnet [89]. This methodology is promising for enhancing the pre-concentration process of analytes and reducing factors such as extraction time and the recovery of the sorbent at the end of each extraction.

In the context of combining magnetic nanoparticles with GBMs for this method, Madej et al. [90] prepared a magnetic composite consisting of Fe_3_O_4_ aggregates grafted onto the surface of rGO for the isolation of seven multiclass pesticides in water samples, followed by HPLC-DAD analysis. The SBSDME method was compared to MSPE. Although SBSDME showed lower recovery values (22–82%) compared to MSPE (20–75%) for the analytes, significant parameters such as good precision, shorter extraction time, ease of coating the bar, and ease of separating the sorbent at the end of extraction/desorption make the SBSDME approach preferable for its practicality.

A study conducted by Vállez-Gomis et al. [91] utilized the same analytical strategy for the determination of residues of ten polycyclic aromatic hydrocarbons (PAHs) in cosmetic samples, followed by analysis using gas chromatography-mass spectrometry (GC-MS). Magnetic cobalt ferrite nanoparticles were grafted onto rGO sheets (CoFe_2_O_4_-rGO) and used as the extraction sorbent after being magnetically deposited onto a neodymium stirring bar. The performance of the method was attributed to the selectivity of the extraction phase, which promoted π-π interactions with the aromatic rings of the analytes and hydrophobic interactions, thus resulting in LOQs ranging from 0.15 to 24.22 ng g^−1^, excellent precision, and enrichment factors between 0.80 and 5.73 for the studied PAHs. Consequently, the method was successfully applied for trace-level investigations in actual cosmetic samples. Approaches such as these have the potential to emerge as promising trends in the functionalization of GBMs with magnetic compounds for SBSDME methodologies.

A notable example is the use of MILs, previously discussed in Section 2.2 of this review. MILs can impart excellent selectivity to GBMs, expanding the range of possible interactions with analytes. This results in a hybrid sorbent with enhanced efficiency, combining the unique properties of graphene compounds with the characteristics of MILs. Such a hybrid sorbent can be applied to isolate a wide range of compounds with diverse traits, showcasing its versatility in analytical applications.

### 3.2. Microextraction by Packed Sorbent 

MEPS stands as another pivotal analytical technique within the realm of solid-based microextraction methods. It was pioneered in 2004 by Abdel-Rehim, drawing upon conventional SPE principles [92]. In MEPS, a packed sorbent (±2 mg) is housed within a syringe, either as a plug or as a cartridge between the barrel and the needle [93,94]. Moreover, the system’s format primarily comprises two key components: the MEPS syringe and the MEPS cartridge, also referred to as the barrel insert and needle (BIN). The packed sorbent within the BIN section is pivotal in interacting with the target, significantly influencing the method’s analytical performance [95]. This analytical approach offers significant advantages by integrating sample extraction, pre-concentration, and clean-up into a single methodology. Therefore, this consolidation enhances the sample throughput for large-scale analyses, streamlining the analytical process and improving efficiency [93,96]. Since the analytical performance of the method is intricately linked to the sorbent phase, the evolution of GMBs has greatly enhanced MEPS applications, ushering in new horizons for this miniaturized approach.

Jordan-Sinisterra and Lanças [54] reported the synthesis of an ionic liquid supported on silica, functionalized with graphene oxide (ILz/Si@GO) as a sorbent phase for MEPS extraction of pesticides in coffee samples, followed by gas-chromatography-tandem mass spectrometry (GC-MS/MS). The authors demonstrated the application of the covalent bonding method, employing N, N′-dicyclohexylcarbodiimide (DCC) as the coupling agent to anchor the IL onto the modified silica. Characterization assays of the synthesized material revealed irregularly shaped particles of uniform size, affirming the successful deposition of IL particles onto GO leaves. Moreover, adsorption experiments indicated a rise in adsorption at 100 µg mL^−1^, reaching saturation at 200 µg mL^−1^. Additionally, the authors stated a substantial enhancement in the adsorption properties of the final sorbent with the application of the IL in the ILz/Si@GO composite. The final application of the sorbent in the MEPS procedure achieved recoveries ranging from 35 to 97%. Although the pesticides aldrin and endosulfan sulfate weren’t detected at quantifiable levels, their identification through the proposed method underscores their persistence in the environment. Such a finding highlights the significance of the developed MEPS sorbent featuring the ILz/Si@GO sorbent phase as a valuable analytical tool for pesticide environmental monitoring.

The rGO sorbent phase was reported by Ahmadi et al. [97] for the MEPS extraction of local anesthetics in plasma and saliva samples with later LC-MS/MS analysis. The study highlighted the exceptional performance of the rGO phase in mitigating matrix effects, eliminating interference peaks at the retention times of target analytes. Notably, the reported method achieved recovery values ranging from 97.26% to 106.83% in plasma and 95.21% to 105.83% in saliva, with LOQ values within the nanomolar range (nmol L^−1^). An outstanding feature of the reported rGO sorbent was its reusability in MEPS extraction. The material demonstrated remarkable durability, enduring over 100 extractions without compromising the analytical performance of the method.

Karimiyan et al. [98] detailed the synthesis and application of a polyacrylonitrile/graphene oxide (PAN/GO) sorbent for microextraction by MEPS, followed by LC-MS/MS analysis of anesthetic drugs (lidocaine, prilocaine) and their metabolites (2,6-xylidine, o-toluidine). The optimization of MEPS revealed that pH significantly influences the interaction of target analytes with the sorbent phase. A basic pH enhances the distribution of the analytes in the sorbent phase, as the target compounds contain amino groups attached to aromatic rings, which are more interactive under alkaline conditions. The obtained LOQ ranged from 2.0 to 10 nmol L^−1^, while the recovery values ranged from 91% to 111%. The authors emphasized that the proposed method was suitable for monitoring drugs in biological samples at low concentration levels, which could be crucial for forensic analysis.

One common challenge encountered when GBMs are applied in MEPS applications is the generation of overpressure within the MEPS syringe due to material obstruction [15]. Addressing this issue, Maciel et al. [99] introduced graphene oxide supported on silica (GO-Sil) to extract tetracyclines from milk samples, followed by LC-MS/MS analysis. Optimization of the method revealed that sampling and elution cycles were the most influential parameters affecting the analytical performance of MEPS. The analytical assessment of the method yielded LOQs ranging from 0.05 to 0.9 µg L^−1^. This exceptionally low LOQ value was instrumental in analyzing 11 commercial samples, detecting traces of tetracyclines in only two. Despite the method’s capability to detect tetracycline traces, the levels observed were within regulatory limits, affirming the efficacy of the GBM sorbent in MEPS applications.

GMBs have found wide application in MEPS across various matrices and analytes. However, there remains a noticeable gap in recent studies that thoroughly explore the potential of these sorbents in MEPS. From the author’s critical perspective, scientific endeavors still need to introduce new graphene-based sorbents into MEPS, particularly with recent applications. Furthermore, there is a pressing need to explore the automation and semi-automation potential of MEPS applications to develop new green analytical strategies and transcend the boundaries of traditional offline methods. By integrating GBMs with automated or semi-automated MEPS alternatives, this miniaturized technique can be elevated to new heights, opening up numerous potential applications.

### 3.3. Pipette-Tip Solid-Phase Extraction and Disposable Pipette Extraction 

Over the years, various forms of miniaturization of conventional SPE have been developed using different devices. PT-SPE is a miniaturized format of SPE, employing a small amount of sorbent packed between two frits or cotton filters within a pipette tip. This tip is then connected to a suitable pipettor or a syringe in more cost-effective setups [13]. Analyte sorption is conducted through sample aspiration and dispensing cycles until partition equilibrium is reached. Generally, the execution of this technique is divided into several steps similar to conventional SPE: (I) conditioning of the sorbent; (II) sorption of analytes (occurring through cycles of aspiration/dispensing); (III) washing of the sorbent to remove sample interferents; (IV) desorption of analytes using an appropriate organic solvent. Advantages over conventional SPE include shorter extraction times, ease of execution, the possibility of on-site extraction, a reduced extraction phase and organic solvent use, and a lower sample volume required [100]. However, since this technique is non-exhaustive, unlike conventional SPE, its performance depends directly on the characteristics of the sorbent used and its specificity for analytes [101].

The unique characteristics of GBMs have led to their successful application in various fields using this technique. For instance, Shen et al. [102] grafted GO nanosheets onto the surface of fibrous silica nanospheres through a water-vapor-induced internal hydrolysis method, aiming at isolating protease inhibitors in yellow catfish, followed by analysis by liquid chromatography-tandem mass spectrometry (LC-MS/MS). Using only 220 mg of the sorbent prepared in a 1 mL pipette tip, the authors achieved LOQs of 0.8–1.6 ng mL^−1^, with analyte recoveries above 70% and good reproducibility. These parameters were attributed to the performance of the sorbent produced, which exhibited a highly rough surface after modification with GO, leading to an increased adsorption capacity of the investigated analytes. Furthermore, the sorbent preparation involved a low amount of organic solvents, with aqueous reaction media and renewable washing solvents (water and ethanol). 

Recently, Zhang et al. [103] modified GO sheets with polydopamine for the extraction of alectinib and its metabolite (M4) in plasma samples using PT-SPE and determination by liquid chromatography with ultraviolet detection (LC-UV). The resulting material exhibited high porosity, increased specific surface area, and was functionalized with functional groups such as amino, hydroxyl, and benzene rings derived from polydopamine. As a consequence, low LOQ values (13.1–16.1 ng mL^−1^), excellent method reproducibility (RSDs ≤ 2.8%), and high analyte recovery rates (>80%) were achieved with just 1 mg of the sorbent packed into the extraction device. The porosity of the material helped to mitigate backpressure issues caused by packing, and the performance of the method results indicated high sorbent selectivity for the target compounds, facilitated by its multiple interaction mechanisms, even in complex biological samples.

In a semi-automated approach, Tsai et al. [104] packed 10 mg of microwave-assisted synthesized rGO nanosheets into a 200 µL pipette tip, connecting it to a commercial plastic syringe attached to a dual syringe pump for the extraction of triclosan from environmental water samples and analysis by HPLC-UV. The authors reported that the primary analyte-sorbent interaction mechanism was the π bonds of rGO with the aromatic rings of the analyte, resulting in a reproducible method with recoveries exceeding 90%. This method was described as simple, cost-effective, and environmentally friendly, as it only required 1 mL of conditioning solvent and the same amount for desorption and sorbent regeneration, achieving reusability for up to 20 extraction cycles. Automated or semi-automated PT-SPE strategies like this hold promise in pursuing greener sample preparation methods, as they promote more sustainable laboratory practices while improving operational efficiency and analyst safety, reducing exposure to toxic substances and the risks associated with manual procedures.

Another approach that uses sorbents in pipette tips is DPX, which emerged in 2003. This method employs an extraction device similar to PT-SPE but with a crucial difference: in DPX, the sorbent is not fixed between two filters (frits). Instead, the sorbent remains free inside the pipette tip, allowing more efficient interaction with the sample and analytes. Only a bottom filter, made of polyethylene or glass wool, retains the sorbent phase within the tip. This configuration facilitates handling and enhances the extraction process efficiency, which follows principles similar to those of dispersive solid-phase extraction (DSPE), based on extraction driven by the contact surface of the sorbent with the sample, achieving a partition equilibrium of analytes between phases. The extraction/desorption steps are the same as those used in PT-SPE, with the washing step not mandatory [105]. Once again, as it is a non-exhaustive technique, the affinity of the sorbent for analytes will play a crucial role in the performance of DPX methods.

When it comes to graphene compounds, Oliveira and Lanças [14] recently anchored GO nanosheets onto silica, which was subsequently functionalized with octadecyl silane endcapped to produce SiGOC18end sorbent employed in DPX for the extraction of eleven multi-class herbicides in sugar cane-derived food and beverage samples, followed by determination by LC-MS/MS. The anchoring of GO onto aminopropyl silica was performed to minimize aggregation issues of GO sheets when dispersed in the sample matrix, and the functionalization of this material with C18 was carried out to enhance the analytes’ surface area and adsorption capacity. The end-capping procedure was conducted to replace surface hydroxyl groups with trimethylsilane groups, rendering the sorbent more apolar and capable of providing hydrophobic interactions with the analytes. Using SiGOC18end in this technique could offer LOQs in the 1–25 ng g^−1^ range for the analytes among the investigated samples and satisfactory linearity (r^2^ > 0.99) for all matrices. The extraction was completed in six minutes, using 0.68 mL of organic solvents, 0.8 mL of sample, and 10 mg of extraction phase.

Until today (May 2024), no further records involving graphene derivatives in DPX methods have been found. One contributing factor may be attributed to the two-dimensional structure of its nanosheets, which could potentially interfere with the dispersive adsorption/desorption process within the pipette tip. However, the advantages of this technique, when combined with the possibility of anchoring and functionalizing these phases with various materials (such as silica derivatives, ILs, and DESs), offer significant potential. These combinations can improve the material structure, address device clogging and particle aggregation issues, and improve selectivity. Consequently, they present many opportunities for future research, providing more efficient and sustainable methods for sample preparation.

### 3.4. Dispersive Micro Solid-Phase Extraction 

Aligned with introducing alternative technologies to conventional SPE, d-µ-SPE emerged as a potential analytical approach for sample preparation [106]. This miniaturization strategy involves dispersing the sorbent into the sample matrix to enhance the kinetic interaction between the sorbent particles and the target analyte(s) [107,108]. The close contact between the sorbent particles and the analytes creates an advantageous condition reflecting improved extraction achievement. The enhanced adsorption capacity of this method relies on retaining matrix components while the analyte remains in the liquid phase, achieved through the introduction of the dispersed sorbent [109]. Given the critical role of the dispersion sorbent in enhancing analytical performance, evaluating the assisted dispersion approach primarily involves studying the utilization of external energy sources (such as mechanical stirring) or chemicals [110]. Employing GBMs as dispersion sorbents in d-µ-SPE has proven to be a versatile strategy for tackling the analysis of complex matrices [21].

Recently, Feist [111] outlined the utilization of GO nanosheets in conjunction with complexing reagents such as neocuproine or batocuproine as the dispersion sorbent for assessing trace-level metal ions in food samples, followed by inductively coupled plasma-optical emission spectrometry (ICP-OES). According to the results, the utilization of both neocuproine and batocuproine reagents enhanced the adsorption of GO by forming cationic complexes with metal ions. Furthermore, a significant achievement was made regarding the preconcentration factor of the GO/neocuproine and GO/batocuproine systems. Both sorbents exhibited preconcentration factors ranging from 10 to 100 and 20 to 200, respectively. Moreover, LOQ values ranged from 0.035 to 0.84, with recovery values over 90% for evaluating heavy metals in food. The developed method was demonstrated as a promising analytical tool for assessing heavy metals in food matrices. 

Another heavy metal detection was proposed by Greda et al. [112] focusing on Cadmium (Cd), using a d-µ-SPE protocol with a GO dispersive sorbent in rose dry wine samples, analyzed by solution anode glow discharge optical emission spectrometry (SAGD-OES). The authors thoroughly investigated the sorption of Cd in comparison to common ions found in the matrix, such as Na^+^, K^+^, Mg^2+^, and Ca^2+^. The results revealed the remarkable adsorption capability of the GO phase, attributed to its functional groups (e.g., –COOH, –OH, =O, and epoxy) acting as electron pair donors, forming strong covalent bonds with metal ions. While most ions were adsorbed onto the GO phase, the analysis emphasized the superior affinity of the GO sorbent towards Cd ions over alkali and alkaline metals. This observation suggests that the strength of covalent bonds with d-block metals like Cd surpassed that of other ions, indicating the GO sorbent’s pronounced selectivity towards Cd ions.

While adopting GBMs as dispersion sorbents has yielded significant advantages for d-μ-SPE, a notable drawback persists: their tendency to aggregate in such applications [110]. This aggregation tendency can diminish analyte adsorption, reducing the available contact area between the sorbent and analyte. As demonstrated in this review, using biosorbents like chitosan can mitigate this aggregation trend, offering a promising solution to enhance the efficacy of d-µ-SPE methodologies. As evidenced by literature examples, biosorbents have already found application in d-µSPE methodologies. For instance, Nakhonchai et al. [113] utilized green hairy basil seed mucilage biosorbent for d-µ-SPE extraction of tetracyclines in bovine milk. This study showcased the biosorbent’s ability to enhance contact between sorbent and analytes, resulting in remarkable recovery values ranging from 83.1% to 109.9%. Moreover, there’s a growing emphasis on developing environmentally friendly biosorbents based on GBMs. This innovation holds the promise of opening new avenues for d-µ-SPE applications, leveraging graphene-based sorbents’ unique properties to enhance extraction efficiencies further.

### 3.5. Magnetic Solid-Phase Extraction 

MSPE is a dispersive extraction technique that resembles the principles of DSPE (and d-µSPE), differing in the type of sorbent used, which in this case consists of magnetic particles. The sorbent comes into contact with the aqueous sample medium, and through an appropriate agitation rate, the analytes are retained in the magnetic phase. Subsequently, the sorbent is separated by applying an external magnetic force to the vial (usually a magnet) and placed in contact with an organic solvent for the desorption process of the compounds, which is performed by ultrasound or agitation. After this step, the sorbent is separated again, and the extract is collected for instrumental analysis [21]. Since this technique also represents a miniaturization of SPE, several advantages are attributed to MSPE, such as eliminating the need for extraction devices like cartridges or syringes. This elimination helps prevent issues like backpressure and clogging, allowing for the use of particles with different physical characteristics [114].

GBMs in this technique have proven very effective due to their relatively high surface area and capacity for extracting organic molecules. The magnetic nanoparticles most commonly attached to GO/rGO sheets in various studies in the literature are magnetite (Fe_3_O_4_), which are deposited on the surface of these materials through electrostatic interactions [114]. Thus, the applications are diverse, as demonstrated in the study by Akamine, Medina, and Lanças [17], where a GO-Fe_3_O_4_ nanocomposite was synthesized for the extraction of gingerols from foods, supplements, and ginger-derived beverages using MSPE and analysis by UHPLC-MS/MS. When compared to other sorbents, such as pure GO, GO@SiO_2_, or commercial C18 particles, the prepared GO-Fe_3_O_4_ sorbent showed equal or superior adsorption capacity for the investigated compounds, standing out for its practicality in being used in the MSPE technique. Consequently, 8 mL of sample was extracted with only 10 mg of the sorbent, resulting in enrichment factors between 9.9 and 30.8 with LOQs of 5 μg L^−1^ for the investigated analytes. The reusability of GO-Fe_3_O_4_ was determined to be 10 cycles without any loss in extraction efficiency. In another study, Kalaboka and Sakkas [115] evaluated the performance of two magnetic sorbents: graphene oxide modified with magnetite particles (Fe_3_O_4_@GO) and magnetite anchored on aminopropyl silica particles functionalized with C18 (Fe_3_O_4_@SiO_2_@C18) for the extraction of 33 emerging multiclass contaminants from various groups in wastewater samples, with determination by LC-Orbitrap MS. The authors found that the GO-based sorbent exhibited a better affinity for more polar compounds due to its hydrophilic functional groups, such as hydroxyl and carbonyl. For 19 of the investigated analytes, using 15 mg of Fe_3_O_4_@GO, the method achieved recoveries above 50% for effluent and tap water samples, with LOQs ≥ 1.2 ng L^−1^ and a reusability of 10 cycles. Both studies demonstrate the high applicability of magnetically modified GBMs for the clean-up and pre-concentration of organic analytes in complex samples.

Mohammadi et al. [116] recently introduced a novel separable sorbent composed of magnetic calcined layered double hydroxide onto graphene oxide (MgO/MgFe_2_O_4_/GO) for efficient extraction of anionic food dyes from water samples, coupled with a straightforward ultraviolet-visible detection (UV-vis) method. In their synthesis, GO and MNPs were amalgamated in methanol as the reaction medium, stirred for 4 h, and dried in an oven at 60 °C. Characterization assays demonstrated a porous and high-surface material essential for the application in actual samples. The LOD and LOQ for the anionic food dyes ranged from 0.167 to 0.14 mg L^−1^ and 0.55 to 0.47 mg L^−1^, respectively. The recovery values for water, saffron, and soft drink samples ranged from 93% to 107%. The proposed graphene-based magnetic sorbent demonstrated exceptional efficacy in extracting anionic food dyes from water samples, offering a versatile and cost-effective analytical approach.

However, to enhance the affinity of graphene sorbents modified with magnetite particles, other materials can anchor and/or functionalize these sorbents, creating hybrid materials. This process improves material performance during the MSPE extraction step, resulting in a more homogeneous dispersion in the aqueous medium and enhanced selectivity for the compounds of interest, achieving a quick and efficient equilibrium. In this line of research, Cao et al. [117] anchored magnetite particles modified with TEOS and APTES (Fe_3_O_4_@SiO_2_-NH_2_) onto graphene oxide, followed by successive in-situ functionalization with β-cyclodextrin and an ionic liquid composed of a cation with an imidazole ring and an anion anthraquinone (VOIm^+^AQSO_3_^−^), resulting in Fe_3_O_4_@SiO_2_/GO/β-CD/IL. This composite was employed in an MSPE method for extracting seven plant regulators from vegetable samples with determination by LC-MS/MS. Utilizing 60 mg of sorbent, equilibrium extraction was achieved within 5 min, leveraging a range of interaction mechanisms inherent in its structure, including π-π stacking, hydrogen bonding, stronger hydrophobic interactions, host-guest inclusion complex formation, and electrostatic interactions. Furthermore, recovery percentages exceeding 80% were attained, along with excellent precision and low relative standard deviation values (≤10.4). The method exhibited outstanding performance, with LOQs ranging from 0.03 to 0.58 μg kg^−1^. 

Recently, Gong and Liu [118] conducted a multi-step synthesis using glutaraldehyde as a crosslinker to modify silica-coated Fe_3_O_4_ with GO. They then functionalized it with chitosan and β-CD for extracting four bisphenols from environmental water and food samples, followed by analysis by LC-FLD. This process resulted in a high surface area (91.83 m^2^ g^−1^), facilitating compound extraction. Adsorption mechanisms involved hydrogen bonding, electrostatic interactions, π-π stacking, and hydrophobic interactions mediated by β-CD. Employing 50 mg of the adsorbent, the method achieved low LOQ levels (0.03 µg L^−1^), with recovery percentages above 80% for the investigated bisphenols and possibly recycling the sorbent up to five times. As discussed, combining MSPE methods with magnetic sorbents containing GBMs, when combined with other materials, enables obtaining more selective phases and more efficient extraction methods. This integration enhances the selectivity and efficiency of extraction processes, reducing the need for large amounts of extraction phase and, consequently, eluent, significantly improving the quality of the results.

## 4. Concluding Remarks and Future Trends

This review offers a comprehensive discussion of the trends in the preparation and application of GBMs, providing an extensive overview of the literature from 2018 to May 2024. As demonstrated, GBMs, including GO and rGO, boast modification possibilities owing to their unique physicochemical properties. Notably, the presence of functional oxygen groups renders them ideal starting materials for developing novel and enhanced sorbent phases. Novel graphene-based sorbents encompass modifications with silica derivatives, ionic liquids, magnetic materials, molecularly imprinted polymers, biomaterials, and deep eutectic solvents. Sorbent materials obtained through these synthesis approaches hold significant promise for different applications. Furthermore, leveraging graphene’s unique physicochemical properties, such as its high surface area and π-π interactions, in combination with these anchored materials, introduces distinct advantages over traditional sorbent materials, thereby enhancing the utility of these sorbents.

While synthesizing graphene-based sorbents offers limitless possibilities, the continued reliance on non-green synthesis methods remains a significant drawback. Most reported methods still involve large solvent volumes, generating substantial chemical residues, posing environmental harm, and compromising human safety. Another critical challenge in graphene-based synthesis is the tendency of 2D structures, such as GO, to form irregular aggregations or self-stacks. Such a drawback directly impacts the specific surface area, consequently affecting the adsorption capability of these materials. To overcome this limitation, it is crucial to suggest using anchoring materials capable of effectively infiltrating between graphene layers to mitigate the aggregation effect. As demonstrated in this study, various materials have been reported in the literature, such as CS, ILs, DESs solvents, and silica derivatives. These materials are primarily applied to mitigate the aggregation drawback of graphene-based materials in microextraction protocols. Incorporating these materials also shows promise for increasing the specific surface area of graphene-based materials and enhancing their recognition capacity.

Moreover, some critical green aspects of GBMs primarily hinge on three main points: (I) biodegradability, (II) reusability, and (III) potential for automation in application methods. Regarding the first aspect, there is a recent trend toward fabricating biodegradable sorbents, representing an eco-friendly concern, especially when these phases are discarded after their useful lifetime. The synthesis of graphene-biosorbents has predominantly centered on utilizing CS, CDs, and DESs as anchoring materials to graphene phases. Besides enhancing environmental consciousness, applying these materials has also bolstered the physicochemical properties of GBMs, opening up new horizons for their application. On the other hand, there is a pressing need to explore new materials, such as agarose, alginate, and cellulose, in synthesizing graphene-biosorbents. Introducing new graphene biosorbents can expand the scope of graphene towards greener analytical approaches, paving the way for novel advancements in the scientific field of sample preparation.

Furthermore, regarding item II, the reusability of the sorbent is crucial for ensuring sustainability. Although the most common protocols for synthesizing GBMs typically involve large volumes of organic solvents and, in some cases, toxic reagents, exploring greener methodologies for obtaining these materials—such as deriving them from biomass or growing graphene from living organisms—appears to be an excellent initiative. Additionally, since miniaturized sample preparation techniques use small amounts of sorbent, investigating their recycling rates is advantageous whenever possible. This reduces the need for repeated syntheses, minimizes waste generation, and enhances the method’s sustainability.

Finally, concerning item III, many applications still rely on conventional offline methodologies despite the significant advantages of using graphene-based sorbents in miniaturized methods, such as minimal organic solvent use and small sample sizes. Focused efforts to propose and explore the automation potential of classical miniaturization methods can significantly advance green analytical practices. Key benefits include higher analytical throughput and reduced human error, thereby enhancing the analytical performance of the process. From the author’s perspective, integrating graphene-based sorbents with miniaturization strategies holds excellent promise for future applications. Furthermore, these enhanced analytical strategies can open new avenues for evaluating diverse analytes and addressing the challenges of analyzing complex matrices, paving the way for a greener analytical future with graphene applications.

## Figures and Tables

**Table 1:** Studies on the application of hybrid graphene-based materials in microextraction techniques.

Modifier Material	Type of GBM	Analyte	Matrix	Microextraction Technique ^1^	Recovery (%)	Reusability Rate (cycles)	Ref.
**Silica Derivatives**	GO@SiO_2_	Benzylpenicillin, Cefalexin, Cefoperazone, and Ceftiofur	Wastewater	Column-switching	>60	100	[28]
	GO@SiO_2_	Carbamazepine, Citalopram, Desipramine, Sertraline and Clomipramine	Urine	Multidimensional LC-MS/MS	-	250	[29]
	SiGO-C18	Simazine, Atrazine, Carbofuran, Tebuthiuron, Diuron, Ametryn, Clomazone, Thiacloprid, Hexazinone, and Imidacloprid	Sugarcane spirits	In-tube SPME	>80	-	[30]
	SiGO-C18	Aflatoxins G2, G1, B2 and B1	Food	PT-SPE	>70	10	[31]
	Si@GO@βCD	Daidzein, Genistein, Formononetin and Biochanin A	Urine	Online SPME-LC	-	200	[23]
**Ionic Liquids**	IL-TGO	Fipronil	Chicken eggs	PT-SPE and DSPE	>90	15	[13]
	IL–CS–GOA	3-bromocarbazole, 2,7-dibromocarbazole and 1,3,6,8-tetrabromocarbazole	Sediment	Glass dropper Extraction	>80	6	[25]
	GO@MIL	Inorganic antimony (Sb III and Sb IV)	Water, Tea And Honey	d-μ-SPE	>97	-	[32]
**Magnetic** **Materials**	MGO	Triazine Herbicides	Fruit and vegetable	MSPE	>70	8	[33]
	MGO@UIO-66	Food colorants	Soft drinks, candies, and pastilles	UA-DSPE	>95	6	[34]
	rGO@MNS	Copper(II)	Environmental waters	SPME	>95	-	[35]
**Molecularly** **imprinted** **polymers**	PDESs-MIP/GO:	Anti-adipogenic drugs	*Solidago decurrens*	CPT-MSPD	>94	-	[36]
	N-GQDs/Fe_3_O_4_ @SiO_2_/IRMOF-1/MIP	Phenylureas	Cucumber, tomato, and radish	d-MSPE	>80	4	[37]
	TPhP-MIPs/GO	Triphenyl phosphate	Environmental water	DI-SPME	>70	110	[38]
	GO/MIP-FA	Organophosphate flame retardants	Environmental water	SPME	>70	110	[39]
**Carbon-based** **biosorbents**	GO/CS	Organic pollutants	Water	SPME	>90	100	[40]
	ACGO	Chlorophenols	Food and environmental samples	TFME	>80	48	[41]
	NiFe_2_O_4_ @GO@ β-CD	Bisphenols	Milk and Milk packing	MPSE	>78	12	[42]
	PDMS/GO/β-CD sponge	Lavander essential oil	Lavender	HS-SPME	-	6	[43]
	β-CD@GO@Si	Isoflavones	Soy-based juice	MEPS	>90	50	[24]
**Deep eutectic** **solvents**	GO@DES	Chlorpyrifos, diazinon, Tebuconazole, Deltamethrin, Permethrin, Haloxyfop-methyl, Penconazole, and Cyhalothrin	Zucchini	d-μ-SPE	>70%	-	[22]
	DFG	Hippuric acid and Methylhippuric acid	Urine	PT-SPE	>90%	-	[44]
	MGO@DES	Estrone, 17β-estradiol and 17α-ethinylestradiol	Milk	MSPE	>90%	7	[45]

^1^**Abbreviations: GO@SiO_2_**: graphene oxide supported on aminopropyl silica; **LC-MS/MS**: liquid chromatography-tandem mass spectrometry; **SiGO-C18:** graphene supported on aminopropyl silica with octadecylsilane; **In-tube SPME:** in-tube solid-phase microextraction; **PT-SPE:** pipette-tip solid-phase extraction; **Si@GO@βCD:** graphene oxide supported on aminopropyl silica particles and modified with β-cyclodextrin; **Online SPME-LC:** Online solid-phase microextraction coupled with liquid chromatography; **IL-TGO:** ionic liquid-thiol-graphene oxide composite; DSPE: dispersive solid-phase extraction; **IL–CS–GOA:** ionic liquid-chitosan-graphene oxide aerogel; GO@MIL: magnetized graphene oxide nanoparticles; **d-μ-SPE:** dispersive micro-solid-phase extraction; **MGO:** magnetic graphene oxide; **MSPE:** magnetic solid-phase extraction; **MGO@UIO-66:** magnetic graphene oxide@UIO-66; **UA-DSPE:** ultrasound-assisted dispersive solid-phase micro-extraction; **rGO@MNS:** metal-doped graphene nanostructure; **SPME:** solid-phase microextraction; **PDESs-MIP/GO:** poly(deep eutectic solvents) surface imprinted graphene oxide composite; **CPT-MSPD:** centrifugation-accelerated pipette-tip matrix solid-phase dispersion method; **N-GQDs/Fe_3_O_4_@SiO_2_/IRMOF-1/MIP:** Fe_3_O_4_@SiO_2_/molecularly imprinted polymer with N-GQDs and IRMOF-1; **d-MSPE**: dispersive magnetic solid-phase extraction; **TPhP-MIPs/GO:** triphenyl phosphate molecularly imprinted polymers immobilized on graphene oxide; **DI-SPME**: direct immersion solid-phase microextraction; **GO/MIP-FA:** GO-based surface molecularly imprinted polymeric fiber array; **GO/CS:** graphene oxide-chitosan; **ACGO:** agarose/chitosan/graphene oxide; TFME: thin film microextraction; **β-CD@GO@Si:** β-Cyclodextrin, coupled to graphene oxide supported on aminopropyl silica; **MEPS:** microextraction by packed sorbent; NiFe_2_O_4_ @GO@ β-CD: β-cyclodextrin-functionalized magnetic graphene oxide; **HS-SPME:** headspace solid-phase microextraction; **PDMS/GO/β-CD sponge:** polydimethylsiloxane/graphene oxide/β-cyclodextrin sponge; **GO@DES:** GO nanoparticles modified with a deep eutectic solvent; **DFG:** deep eutectic solvent functionalized graphene oxide; **MGO@DES:** magnetic graphene oxide modified with deep eutectic solvent.
